# The Effect of Dopamine Antagonist Treatment on Auditory Verbal Hallucinations in Healthy Individuals Is Clearly Influenced by *COMT* Genotype and Accompanied by Corresponding Brain Structural and Functional Alterations: An Artificially Controlled Pilot Study

**DOI:** 10.3389/fgene.2019.00092

**Published:** 2019-03-06

**Authors:** Chuanjun Zhuo, Yong Xu, Li Zhang, Rixing Jing, Chunhua Zhou

**Affiliations:** ^1^ Department of Psychiatric-Neuroimaging-Genetics and Comorbidity Laboratory (PNGC-Lab), Tianjin Mental Health Centre, Mental Health Teaching Hospital of Tianjin Medical University, Tianjin Anding Hospital, Tianjin, China; ^2^ Department of Psychiatry, College of Basic Medical Science, Tianjin Medical University, Tianjin, China; ^3^ Department of Psychiatry, First Hospital/First Clinical Medical College of Shanxi Medical University, Taiyuan, China; ^4^ MDT Center for Cognitive Impairment and Sleep Disorders, First Hospital of Shanxi Medical University, Taiyuan, China; ^5^ Department of Psychiatry, Institute of Mental Health, Jining Medical University, Jining, China; ^6^ GHM Institute of CNS Regeneration, Jinan University, Guangzhou, China; ^7^ Department of Pattern Recognition, China National Key Laboratory, Institute of Automation, Chinese Academy of Sciences, Beijing, China; ^8^ Department of Pattern Recognition, University of Chinese Academy of Sciences, Beijing, China; ^9^ Department of Pharmacy, The First Hospital of Hebei Medical University, Shijiazhuang, China

**Keywords:** *COMT* genotypes, auditory verbal hallucinations, dopamine antagonists, brain alterations, health individuals

## Abstract

Few studies have been conducted to explore the influence of the catechol-o-methyltransferase (*COMT*) genotype on the severity of and treatment efficacy on auditory verbal hallucination (AVH) symptoms in healthy individuals with AVHs (Hi-AVHs). We hypothesized that the efficacy of dopamine antagonist treatment on AVHs in Hi-AVHs may be influenced by their *COMT* genotype and may be accompanied by corresponding brain alterations. To preliminarily investigate and test our hypothesis in an artificially controlled pilot study, we enrolled 42 Hi-AVHs as subjects and used magnetic resonance imaging and genetic methods to explore the basis brain features to investigate whether the efficacy of dopamine antagonist treatment on AVHs in Hi-AVH subjects was influenced by their *COMT* genotype or not. We found that *COMT*-met genotype subjects’ treatment response was better than that of *COMT*-val subjects. Although *COMT*-met genotype subjects demonstrated an increase in global functional connectivity density (gFCD) but no difference on gray matter volume (GMV) compared to COMT-val genotype subjects at baseline, notably, we found that both groups demonstrated gFCD and GMV reduction after treatment, but the reduction was more widespread in *COMT*-met genotype subjects than in *COMT*-val genotype subjects. This is the first study to report that Hi-AVH subjects’ baseline brain functional features are influenced by their *COMT* genotypes and that the *COMT*-met genotype subjects exhibit better responses to dopamine antagonists but have more widespread GMV and gFCD reduction than subjects with the *COMT*-val genotype. Despite several limitations, these findings may provide auxiliary information to further explain the mechanisms of AVHs and provide a clue for scholars to further explore specific treatment targets for AVHs in Hi-AVH subjects or in schizophrenia patients.

## Introduction

According to the previous findings, even according to the strictest criteria, 0.7% of the general population has experienced auditory verbal hallucinations (AVHs); these subjects are usually called healthy individuals with AVHs (Hi-AVHs) (Johns et al., 2004; Sommer et al., 2010; Upthegrove et al., 2016). Many hypotheses of AVHs have been established in recent decades; each hypothesis explains AVHs from a different perspective (Jones, 2010; Liemburg et al., 2012; Cho and Wu, 2014; McCarthy-Jones et al., 2014; Northoff, 2014; Wilkinson, 2014; Alderson-Day et al., 2015, 2017; Hugdahl, 2015; Conde et al., 2016; Baumeister et al., 2017; Curcic-Blake et al., 2017; Wilkinson and Fernyhough, 2017). To date, however, no hypothesis has achieved general acceptance (Wilkinson and Fernyhough, 2017). Many studies have proposed that investigating AVHs in Hi-AVHs subjects can provide important information to help clarify the mechanisms of AVHs (Jones, 2010; Hugdahl, 2015; Wilkinson and Fernyhough, 2017).

The catechol-o-methyltransferase (*COMT*) genotype influences brain functional (including auditory processing, which is highly related to AVHs) and dopaminergic alterations both in healthy people and in patients with schizophrenia (Lu et al., 2007; Kang et al., 2010; Gothelf et al., 2011; Edgar et al., 2012; Tian et al., 2013a,b; Li et al., 2015; Steiner et al., 2018) and the efficacy of dopamine antagonists in patients with schizophrenia (Olgiati et al., 2009; Sagud et al., 2010; Huang et al., 2016). These previous findings converge to indicate that the reciprocal interactions between *COMT* genotype, dopamine levels, and structural and/or functional alterations in the human brain are related to mental disorder symptoms, such as AVHs (Lu et al., 2007; Kang et al., 2010; Sagud et al., 2010; Gothelf et al., 2011; Edgar et al., 2012; Tian et al., 2013a,b; Li et al., 2015; Huang et al., 2016; Steiner et al., 2018).

*COMT*-met genotype patients with schizophrenia respond more strongly than *COMT*-val genotype patients to dopamine antagonists. In particular, patients with positive symptoms respond more strongly to dopamine antagonists in the former group than in the latter, and this response is associated with corresponding brain structural and functional alterations (Edgar et al., 2012; Lei et al., 2015; Gong et al., 2016). AVHs are the classic positive symptoms (Tandon, 2013; Reed et al., 2018). However, no study has reported the efficacy of antipsychotics on AVHs in schizophrenic patients. Most studies refer to AVHs as part of the positive symptom cluster and do not report them as a distinct category (Olgiati et al., 2009; Sagud et al., 2010; Huang et al., 2016). To the best of our knowledge, only one study has reported that the efficacy of transcranial direct current stimulation (tCDS) as a supplement to antipsychotic treatment of AVHs is weaker in schizophrenia patients with the *COMT*-met genotype than in those with the *COMT*-val genotype (Chhabra et al., 2018). A recent systematic review reported that antipsychotics can improve AVHs in patients with borderline personality disorder (Slotema et al., 2018). This study indicates the feasibility of exploring the efficacy of antipsychotics on AVHs. Exploring the pathological features of Hi-AVH subjects and the efficacy of antipsychotic treatment on them can provide new fundamental information for exploring the mechanisms of AVHs in patients with schizophrenia. The recruitment of Hi-AVHs subjects can prevent many confounding factors, such as other positive symptoms.

To the best of our knowledge, few studies have been conducted to explore the influence of the *COMT* genotype on the severity of AVH symptoms in healthy individuals with AVHs, to explore the relationship between brain structural and functional alterations and the COMT genotype, or to explore the efficacy of dopamine antagonists on AVHs in Hi-AVH subjects in order to explore the corresponding brain structural or functional alterations that accompany the efficacy of treatment. *We proposed a hypothesis that the* efficacy *of dopamine antagonist treatment on AVHs symptoms in Hi-AVHs subjects is influenced by the COMT genotype and is accompanied by structural and functional brain alterations.*

In the present pilot study, we adopt genotyping, functional connectivity density mapping, and statistical parametric mapping (SPM) techniques to explore the influence of the *COMT* genotype on AVH symptoms in Hi-AVH subjects and explore the influence of the *COMT* genotype on the efficacy of dopamine antagonist treatment on AVHs in Hi-AVH subjects and the accompanying brain structural and functional alterations.

## Samples and Methods

### Samples

For the present pilot study, we recruited by advertisement in 1,000 communities (total resident population greater than 200,000) to enroll Hi-AVH volunteers from 1,1,2016 to 6,31,2018. In accordance with previous studies (Haddock et al., 1999; Xu et al., 2012; Dollfus et al., 2018), we adopted the traditional Auditory-Verbal Hallucinations Rating Scale (AHRS) extracted from the Psychotic Symptom Rating Scales (PSYRATS) to assess the severity of the AVH symptoms. We enrolled 300 healthy people with diagnosed AVHs. Among them, only 115 subjects reported that they had suffered mental distress caused by the AVHs and volunteered to accept treatment. None of the subjects who participated in the treatment processing reported psychiatric positive family history, childhood trauma, abuse, or any other negative life events. Simultaneously, none of the subjects achieved the diagnostic criteria of any clearly mental disorders according to DSM-IV, depending on the assessment by two senior psychiatrists according to the SCID-NP semistructured interview (Phillips et al., 2009). The Tianjin Anding Hospital ethics review board approved this study. All patients provided written consent. The assessments were carried out in compliance with the Declaration of Helsinki guidelines and approved by the institutional ethics committee.

### Methods

#### Genotyping

The genotypes were grouped by allele dominance according to the available literature (Chhabra et al., 2018). Blood collection and genotyping were performed as previously reported (Chhabra et al., 2018). In brief, 5 ml of peripheral blood was collected in K_2_EDTA-treated vacutainers (Becton & Dickinson, NJ, USA), and genomic DNA was extracted using commercial spin columns (Qiagen, Inc., Limburg, the Netherlands). The quality of extracted DNA was determined by UV spectrophotometry (Thermo Scientific, Waltham, MA, USA). Then, the genomic DNA was subjected to COMT genotyping at rs4680 using the TaqMan 5′ nuclease allelic discrimination assay. The genotyping was performed by real-time polymerase chain reaction (PCR) in a 96-well plate (StepOne Plus™ Real-Time PCR Systems, Applied Biosystems) with predesigned, commercially made primers and allele-specific minor groove binding probes (FAM and VIC; Applied Biosystems, Foster City, CA, USA) in a reaction volume of 10 μl (10 ng of genomic sample DNA, assay mix, and PCR Universal Master Mix with AmpErase^®^ Uracil-DNA Glycosylase) as follows: 60°C for 30 s, and 95°C for 10 min, followed by 50 cycles of 92°C for 15 s and 60°C for 90 s. PCR was performed in duplicate with both positive and negative controls. In accordance with previous studies, *COMT*-met is the subjects with *COMT*-met/met, while *COMT*-val is the subjects with *COMT*-met/val and *COMT*-val/val (Kang et al., 2010).

#### Magnetic Resonance Imaging (MRI) Data Acquisition

All MRI data were obtained on a 3.0-tesla MR system (Discovery MR750, General Electric, Milwaukee, WI, USA). Tight but comfortable foam padding was used to stabilize head position, and earplugs were used to reduce scanner noise during image acquisition. A sagittal 3D T1-weighted brain volume sequence with 188 sagittal slices was performed with the following scan parameters: repetition time (TR) = 8.2 ms; echo time (TE) = 3.2 ms; inversion time (TI) = 450 ms; flip angle (FA) = 12°; field of view (FOV) = 256 mm × 256 mm; matrix = 256 × 256; slice thickness = 1 mm, no gap. Resting-state functional MRI (fMRI) scans were performed using a gradient-echo single-shot echo-planar imaging sequence with scan parameters of TR/TE = 2000/45 ms, FOV = 220 mm × 220 mm, matrix = 64 × 64, FA = 90°, slice thickness = 4 mm, gap = 0.5 mm, 32 interleaved transverse slices, and 180 volumes. During fMRI scans, all subjects were instructed to keep their eyes closed, to relax, to move as little as possible, to think of nothing in particular, and not to fall asleep.

#### fMRI Data Preprocessing

Resting-state fMRI data were preprocessed using SPM8 (http://www.fil.ion.ucl.ac.uk/spm). The first 10 volumes for each subject were discarded to allow for scanner stabilization and the participants’ adaption to the scanning environment. The remaining volumes were preprocessed after a slice-timing correction. All subjects’ fMRI data were within defined motion thresholds (i.e., translational and rotational motion parameters less than 2 mm or 2°). Several nuisance covariates (six motion parameters and average BOLD signals of the ventricles and white matter) were regressed out of the data. Framewise displacement (FD), which indexes volume-to-volume changes in head position, was also calculated. If the FD of the specific volume was over 0.5, spike volumes were also regressed out. The datasets were bandpass filtered with frequencies ranging from 0.01 to 0.08 Hz. Individual structural images were linearly coregistered to the mean functional image, and the transformed structural images were linearly coregistered to the Montreal Neurological Institute (MNI) space. Finally, the motion-corrected functional volumes were spatially normalized to the MNI space using parameters that were estimated during linear coregistration. The functional images were resampled into 3-mm cubic voxels.

#### gFCD Calculation

We calculated the gFCD of each voxel using an in-house script on the Linux platform as previously reported (Tomasi and Volkow, 2010, 2011). The strength of the functional connectivity between voxels was evaluated using Pearson’s linear correlation with a correlation coefficient threshold of R > 0.6. The gFCD calculation was restricted to voxels in the cerebral gray matter regions using a cerebral gray matter mask. The gFCD at a given voxel x0 was computed as the total number of functional connections, k(x0), between x0 and all other voxels using a “growing” algorithm that was developed on the Linux platform. This processing calculation was repeated for all voxels x0 in the whole brain. To increase the normality of the distribution, we applied grand mean scaling to gFCD by dividing by the mean value of the qualified voxels in the whole brain. Finally, to minimize differences in the functional brain anatomy across subjects, we spatially smoothed the FCD maps with a 6 × 6 × 6 mm^3^ Gaussian kernel.

#### Gray Matter Volume (GMV) Calculation

The GMV of each voxel was calculated using Statistical Parametric Mapping (SPM8; http://www.fil.ion.ucl.ac.uk/spm/software/spm8/). With the standard unified segmentation model, we segmented structural MR images into gray matter (GM), white matter, and cerebrospinal fluid. After an initial affine registration of the GM concentration map into the MNI space using the technique of diffeomorphic anatomical registration through exponentiated Lie algebra, GM concentration images were nonlinearly warped and then converted to a voxel size of 1.5 × 1.5 × 1.5 mm^3^. The nonlinear determinants were first derived from the spatial normalization step and then multiplied by the GM concentration map to obtain the GMV of each voxel. Finally, the GMV images were smoothed with a 6 × 6 × 6 mm^3^ full width at half-maximum Gaussian kernel. The normalized, modulated, and smoothed GMV maps were used for statistical analyses after spatial preprocessing.

#### Statistical Analysis

A two-sample *t*-test was used to compare gFCD between groups in a voxelwise manner with adjustment for age and sex. A familywise error (FWE) method was used to correct for multiple comparisons (*p* < 0.05). If a significant difference between groups was found in the mean gFCD of each cluster, it was extracted for each subject and then used for region of interest (ROI)-based comparison between groups. The possible effect of GMV on global FCD changes was excluded by comparing the GMV of each ROI between groups as an added covariate of no interest. For these ROI-based analyses, the effect size of each comparison was described using Cohen’s d. To further investigate whether the gFCDs were correlated with clinical variables, we used a partial correlation analysis to analyze the relationship of ROI-based analyses with antipsychotic doses of chlorpromazine equivalents, illness duration, and adjusted for age and sex. Partial correlation analyses were also performed to investigate the relationship between AVH scores and gFCD values, adjusted for age, gender, educational level, and antipsychotic dose. The Bonferroni method was used to correct for multiple comparisons (*p* < 0.05).

## Results

### Sample Information

**Figure 1:**
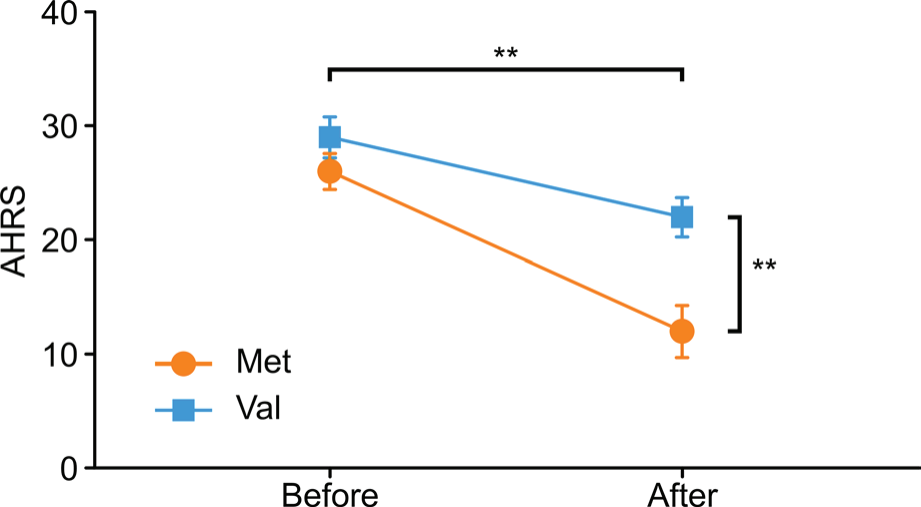
Differential effects of treatment in two groups (note: ** *P* < 0.01).

Ultimately, 34 *COMT*-met subjects and 45 *COMT*-val subjects underwent dopamine antagonist treatment for 6 months. However, the MRI data from only 25 *COMT*-met subjects and 21 *COMT*-val subjects could be used for analysis. We sought to assess accurately how the *COMT* genotype influenced the antipsychotic efficacy and accompanying corresponding brain alterations. We factitiously discarded information from three *COMT*-met subjects and one *COMT*-val subject (a flaw of the present study; please see the section on limitations), preserving only 22 *COMT*-met subjects and 20 *COMT*-val subjects for further analysis to guarantee comparability. All the sociodemographic, genotype, and treatment response information are shown in Table 1. There were no significant group differences in gender, age, educational level, illness duration, or baseline AVH symptom severity. During the treatment, all subjects took risperidone (Johns and Johns, Xi’an Yang-Sen Pharmaceutical Co., Ltd.) as treatment, and their antipsychotic dosages (in chlorpromazine equivalents) ranged from 500 to 100 mg/d. Antipsychotic dosage showed a significant difference between genotypes, with *COMT*-val subjects receiving higher dosages than *COMT*-met subjects. Surprisingly, however, despite largely comparable medication regimens, the efficacy of antipsychotics on AVHs was remarkably different between two groups (Table 1 and Figure 1, Note: ** < 0.001).

**Table 1:** Sociodemographic and clinical characteristics.

	*COMT*-met	*COMT*-val	*t/x^2^*	*P*
Age	24.91 ± 3.58	25.23 ± 3.61	−0.293	0.771
Gender	Male 7 Female 15	Male 10 Female 10	1.437	0.231
Education level (years)	11.91 ± 2.33	11.86 ± 1.81	0.072	0.943
Illness duration (months)	128.73 ± 40.84	129.86 ± 43.69	−0.089	0.929
Antipsychotic dosage (mg/d, chlorpromazine equivalents)	250.40 ± 50.10	300.15 ± 75.50	−2.564	0.014
AHRS score at baseline	28.41 ± 4.25	28.32 ± 5.40	−0.279	0.781
ARHS score after treatment	12.18 ± 4.08	23.14 ± 5.89	6.781	< 0.001
ARHS score at baseline *vs.* ARHS score after treatment	*t* = 8.341 *P* < 0.001	*t* = 2.974 *P* = 0.005		

Note: AHRS: Auditory verbal rating scale.

### gFCD and GMV Differences Between Two Groups at Baseline

**Figure 2:**
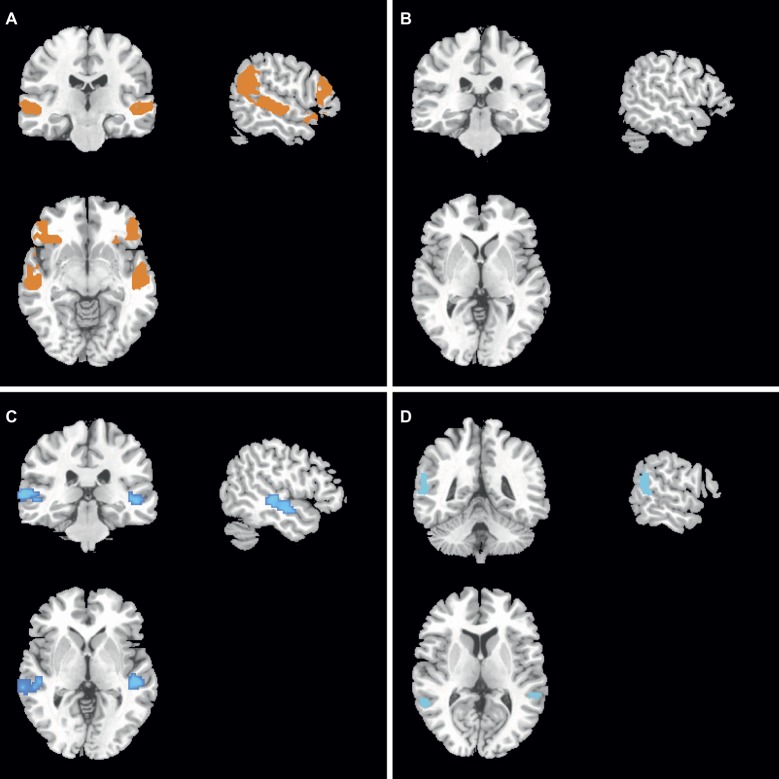
gFCD and GMV differences between two groups.

At baseline, compared to the *COMT*-val genotype subjects, the current study found that *COMT*-met genotype subjects demonstrated higher gFCDs located in the auditory cortex (superior temporal gyrus or Wernicke brain region, *p* < 0.05, corrected with FWE) (Figure 2A). Simultaneously, the ventral lateral prefrontal lobe, which was related to mood regulation, also demonstrated higher gFCD (Figure 2A). However, GMV showed no significant differences between two groups (Figure 2B).

### gFCD and GMV Differences Between Two Groups After Treatment

After treatment, compared to the *COMT*-val genotype subjects, the present study found that *COMT*-met genotype subjects demonstrated lower gFCD in the middle temporal gyrus (Figure 2C). More notably, *COMT*-met genotype subjects’ GMV was also significantly lower in the lateral parietal lobe (Figure 2D).

### gFCD and GMV Differences Before and After Treatment in Each Group

**Figure 3:**
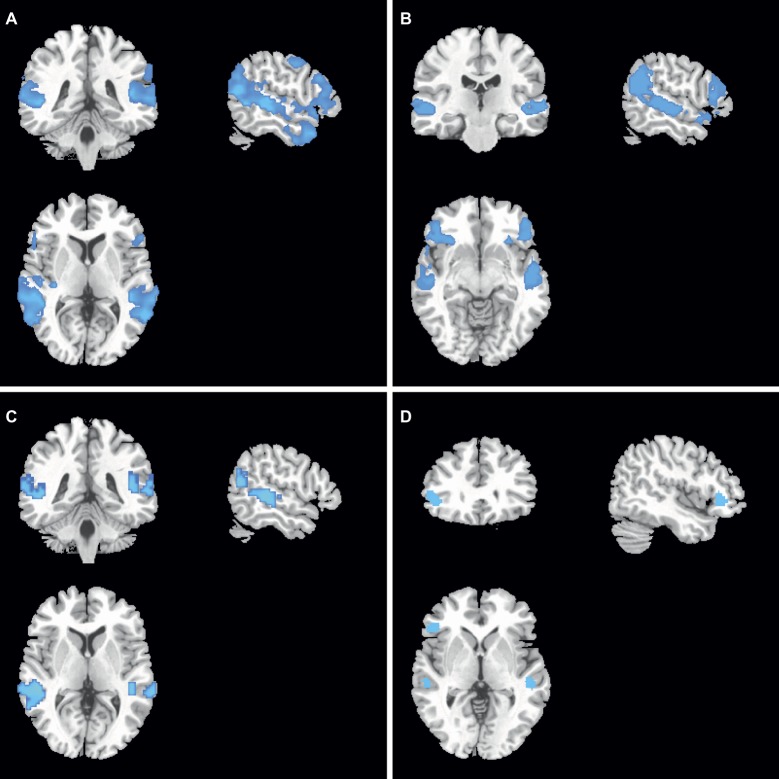
gFCD and GMV differences before and after treatment in each group.

In the *COMT*-met subjects, gFCD was lower in the lateral parietal lobe, dorsolateral prefrontal cortex, posterior superior temporal gyrus, temporal pole, and motor cortex after treatment (Figure 3A), while GMV was lower in the lateral frontal lobe, lateral temporal lobe, and lateral parietal lobe (Figure 3B). However, in the *COMT*-val subjects, gFCD was lower in the posterior superior temporal gyrus and posterior parietal lobe (Figure 3C), and GMV was lower in the superior temporal gyrus and ventral lateral prefrontal lobe (Figure 3D).

### The Association Among gFCD, GMV, and AVHs

In each group, we found no statistical correlations between gFCD and antipsychotic dosage in chlorpromazine equivalents, illness duration, or AVH scores before or after treatment. Similarly, we also did not find any statistical correlations between GMV and antipsychotic dosages in chlorpromazine equivalents, illness duration, or AVH scores before or after treatment.

## Discussion

The present artificially controlled pilot study is the first one to demonstrate that the efficacy of dopamine antagonists on Hi-AVH subjects was influenced by the *COMT* genotype and was accompanied by corresponding brain structural and functional alterations. More importantly, we not only compared treatment efficacy and brain alterations between two groups but also assessed the difference before and after treatment in each group, providing supplementary information to further clarify the pathological features of Hi-AVHs with specific *COMT* genotypes. Although this pilot study has several limitations, it can at least provide a clue to guide further study.

Mounting studies have confirmed that GMV, usually referred to as structural alterations, affects many functional activities and subsequently causes mental and behavioral alterations (Asami et al., 2013; Rogers and De Brito, 2016). gFCD is an index evaluating functional connectivity, and many previous studies have also reported that gFCD indexes informational communication capacity to some extent. gFCD increase indicates that information communication throughout the brain is enhanced and vice versa (Goni et al., 2014; Teodoro et al., 2018). The GMV and gFCD alterations in Hi-AVH subjects after treatment indicate that antipsychotics may normalize structural and functional aberrations of the brain, subsequently alleviating AVH symptoms, although the treatment efficacy is influenced by the *COMT* genotype.

In this pilot study, we found that *COMT*-met Hi-AVH subjects achieved markedly better treatment efficacy than those with the *COMT*-val genotype; correspondingly, brain structural and functional alterations are also more widespread after treatment in the former group than in the latter. However, we did not find any correlation between the gFCD or GMV alterations accompanying AVH alleviation and the dosage of antipsychotics or the duration of illness. More interestingly, we also found that *COMT*-met subjects had a broader scope of brain structural and functional aberrations before and after treatment than *COMT*-val subjects. These aberrant brain regions are involved in multiple types of information processing; for example, the superior temporal gyrus participates in the processing of auditory information, the ventral lateral prefrontal lobe and lateral parietal lobe participate in cognitive information processing, the posterior superior temporal gyrus and posterior parietal lobe are related to language processing, and the temporal pole participates in language processing and multisensory integration (Fan et al., 2014; Xu et al., 2015). These findings indicated that Hi-AVH subjects also had functional aberrations in many brain regions that modulate multiple types of functional activity in the brain, not limited to auditory information processing. More importantly, after treatment, all subjects also demonstrated normalization of hyperfunctional activity, which indicated that GMV was impaired in all subjects. More notably, the GMV reduction before and after treatment was more widespread among *COMT*-met subjects than among *COMT*-val subjects, which indicated that antipsychotics may have caused GMV reduction or the normalization of enlarged GMV before treatment. This complex phenomenon requires further study of genotypically similar normal controls and Hi-AVH subjects to clarify. However, some studies have reported that antipsychotics may cause GMV reduction (Asami et al., 2013; Rogers and De Brito, 2016). As for the normalization of aberrant functional activity by antipsychotic treatment, this finding has been confirmed by many studies (Gong et al., 2016). Therefore, we do not expand on the explanation in this paper. Our data may support the postulation that antipsychotics normalize enlarged GMV before treatment, which has very little support from current available literature. Of course, additional attention and studies are required to confirm this conclusion, since the subjects in our study took antipsychotics for only 6 months.

AVHs in schizophrenia have been reported by many studies from multiple perspectives, from a clinical to a combination of neuroimaging, electrophysiological, and neurotransmitter perspectives (Lu et al., 2007; Kang et al., 2010; Gothelf et al., 2011; Edgar et al., 2012; Steiner et al., 2018). Multiple specific figures are also corroborated by many studies and some are generally accepted to some extent. However, few studies have reported on AVHs in Hi-AVH subjects. Our pilot study also found that AVH symptoms in Hi-AVHs are nearly as severe as in schizophrenia patients, and many subjects desired treatment. Further study is needed to explore specific strategies by which to treat these subjects, especially considering the GMV impairment caused by antipsychotics. In only 6 months of treatment, the GMV decreased at a remarkable speed. One possible explanation is that the GMV in Hi-AVH subjects was enlarged compared with that of healthy controls, while antipsychotics normalized the enlarged GMV, thus causing GMV reduction. However, this postulation is highly speculative and cannot be adequately tested with the current data. Therefore, further study is urgently needed to address this hypothesis.

### Limitations

By listing the flaws of this pilot study, we hope to help other scholars avoid similar weaknesses in their research. This study has at least 12 limitations, which we list in the following paragraphs; we sincerely hope that international scholars will provide constructive comments to guide our subsequent studies.

First, to evaluate the practicability of this study, we adopted many methods to ensure that the subjects could complete the full study. However, despite our best efforts, only 42 subjects with adequate data can be used for the whole analysis. In order to improve the accuracy of investigating the influences of COMT genotype on the treatment efficacy of dopamine antagonist on the AVH symptoms in the Hi-AVH subjects, a long-term cohort study with large sample will be necessary. Given this pilot study, we should rethink our method to assure that large samples are sufficiently enrolled in the study. Second, to explore potentially objective evaluation indices, we discarded four samples that deviated substantially from other samples. The present study was only a pilot study, and we need to strengthen our study methods so that heterogeneous samples can be analyzed. Third, here we adopted the relatively simple indices of gFCD and GMV to explore brain alterations. More precise MRI data analysis methods are currently available, and we should adopt the most advanced method to analyze brain alterations in future studies. Fourth, in this pilot study, we considered only the *COMT* genotype. Other genes, such as *FOX2* gene, *NRG1*, and other newly identified genes, were not examined. We should adopt genomic, transcriptomic, and even proteomic methods for further studies. Fifth, here we used a 3.0-T scanner to acquire MRI data. Currently, a 7.0-T scanner is in use in China. A higher resolution scanner should be applied in future studies to explore the subtle alterations in the brain. Sixth, we did not consider treatment time or reciprocal gene interaction effects, which should be taken into account in further studies. Seventh, an important flaw in this pilot study is that, limited by the condition, at baseline, we did not enroll healthy controls with *COMT*-met and *COM*-val to compare their brain alterations with those of similarly genotyped Hi-AVH subjects. Therefore, with current data, we cannot clarify whether the brain structural and functional alterations exist or not in the Hi-AVH subjects at baseline. However, comparison was performed in one patient before and after treatment in the present study. This self-control comparison may somewhat remedy the study flaw. In future studies, we must solve this problem to achieve a more precise understanding of AVHs-related brain structural alterations in the Hi-AVHs. Eighth, we did not enroll schizophrenia patients with AVHs for comparison, which limits the information that can be gained from the present study. The ninth limitation of this pilot study is that we did not compare cognitive alterations before antipsychotic treatments. To the best of our knowledge, there are no studies reporting any influence of antipsychotics on the cognitive ability of psychiatrically healthy subjects. Conversely, many studies have reported that antipsychotics (including risperidone) have positive effects on cognitive impairments in patients with schizophrenia (Suzuki and Gen, 2012; Desamericq et al., 2014), which indicates that antipsychotics can improve cognitive impairments or, at least, does not impair cognitive ability. Hence, we need to clarify the effect of antipsychotics on the cognitive ability of Hi-AVHs in the future. Tenth, when we found the GMV were lower after six-month treatment, we worried about the influences of the treatment on the subjects’ social and cognitive function. Thus, we adopted Global Assessment of Functioning (GAF) scale (Vaskinn and Abu-Akel, 2018) and Wisconsin Card Sorting Test (WCST) (Westwood et al., 2016) to evaluate each subject, which was used as a remedy method to define whether the dopamine antagonist caused the functional impairment or not in the subject. Fortunately, all subjects scored within the normal range. This problem should be avoided in the future study. More importantly, as mentioned above, the GMV in the subjects receiving treatment decreased so quickly in the Hi-AVH subjects that we must be highly vigilant. According to our pilot study, we suggest that the dopamine antagonist is not the appropriate treatment for Hi-AVH subjects. Eleventh, in this pilot study, we calculated the gFCD both with and without the global signal and found little difference between the two values. As there is no consensus as whether to include the global signal or not in calculating gFCD, we reported the gFCD with the global signal here. Twelfth, as this was a pilot study, we did not compare differences in the demographic and clinical data between the subjects who completed and those who did not complete the full study. In this study, we aimed to observe the influence of the *COMT* genotype on the efficacy of atypical antipsychotics on AVHs in the Hi-AVH subjects. However, we did not genotype *COMT* in the subjects who failed to complete the study, which was a flaw of this study.

## Conclusion

In this artificially controlled pilot study, despite many limitations existed, we report for the first time that *COMT* genotypes influence the functional features of brains and the efficacy of dopamine antagonists on the treatment of AVHs in Hi-AVH subjects. *COMT*-met genotype subjects responded more strongly to dopamine antagonists but also had more serious GMV and FCD reductions than *COMT*-val subjects. Although the design of this pilot study is less than optimal, these findings can at least provide primary information to further explain the mechanisms of AVHs and to help reveal specific targets for the treatment of AVHs in Hi-AVH subjects or in schizophrenia patients.

## Author Contributions

CZhuo and CZhou had full access to all of the data in the study and took responsibility for the integrity of the data and the accuracy of the data analysis. CZhuo and CZhou contributed to the study concept and design. YX, LZ, and RJ participated in acquisition, analysis, or interpretation of data. CZhuo, YX, LZ, and RJ performed statistical analysis. CZhuo and CZhou contributed to administrative, technical, or material support. CZhuo involved in drafting of the manuscript.

### Conflict of Interest Statement

The authors declare that the research was conducted in the absence of any commercial or financial relationships that could be construed as a potential conflict of interest.
